# CT characteristic of early local recurrence after resection of the squamous cell carcinoma: comparison with CT characteristics of granulation tissue at stump site

**DOI:** 10.1186/1470-7330-15-S1-P22

**Published:** 2015-10-02

**Authors:** Hye Jeon Hwang, Mi Young Ki, Chang-Min Choi

**Affiliations:** 1 Department of Radiology, Hallym University College of Medicine, Hallym University Sacred Heart Hospital, Korea; 2 Department of Radiology and Research Institute of Radiology, University of Ulsan College of Medicine, Asan Medical Center, Seoul 138-736, Korea, 86 Asanbyeongwon-Gil, Songpa-Gu, Seoul 138-735, Korea; 3 Pulmonary and Critical Care Medicine, University of Ulsan College of Medicine, Asan Medical Center, Seoul 138-736, Korea, 86 Asanbyeongwon-Gil, Songpa-Gu, Seoul 138-735, Korea; 4 Oncology, University of Ulsan College of Medicine, Asan Medical Center, Seoul 138-736, Korea, 86 Asanbyeongwon-Gil, Songpa-Gu, Seoul 138-735, Korea

## Aim

To compare thin section CT characteristics of the early local tumour recurrence after the resection of squamous cell carcinoma (SCC) with those of the stump deformity or granulation tissue.

## Methods

Twenty nine consecutive patients with local recurrence after definitive SCC operation from April 2006 to September 2012 were included. Pre- and post-operative CT from these patients were retrospectively reviewed and compared with those in age- and gender-matched 29 patients with stump deformity or granulation tissue at stump site after the definitive SCC operation, by two chest radiologists.

## Results

Local recurrence were commonly observed as round/oval shape, peripheral eccentric lesion or central contour bulging lesion on CT, while the stump deformity or granulation tissue were commonly demonstrated as irregular or flat shape, focal wall thickening. The size of suspected soft tissue and the distance between stump staples and suspected soft tissue were significantly different between two groups (median; 19mm and 3mm; 18mm and 0mm, respectively, *p*< 0.001). The univariate analysis showed that the size of soft tissue and the distance between soft tissue and stump site were associated with the predictive factors of local recurrence (*p*< 0.001). On the receiver operating characteristic analysis, the optimal cut-offs of the size of soft tissue and the distance between soft tissue and stump staples were 6mm and 5mm, respectively.

## Conclusion

The proper knowledge of stump recurrence regarding the size and the distance around the stump on CT imaging will help us achieve an early and higher diagnostic rate of recurred SCC.

